# *In situ* sulfuration synthesis of flexible PAN-CuS “flowering branch” heterostructures as recyclable catalysts for dye degradation†

**DOI:** 10.1039/c8ra08293f

**Published:** 2018-12-05

**Authors:** Yin Lu, Yanjie Wang, Shizhong Cui, Weihua Chen, Liwei Mi

**Affiliations:** Center for Advanced Materials Research, Zhongyuan University of Technology Zhengzhou 450007 P. R. China mlwzzu@163.com; College of Chemistry and Molecular Engineering, Zhengzhou University Zhengzhou 450001 P. R. China chenweih@zzu.edu.cn

## Abstract

“Flowering branch”-like PAN-CuS hierarchical heterostructures were *in situ* synthesized through a facile hydrothermal sulfuration growth process on PAN-based fibers prepared by electrospinning. The PAN fibers can serve as a stable flexible support, while CuS flowers assembled from nanosheets can act as reactive materials, showing high performance in the degradation of dyes. Moreover, these heterostructures can be recovered easily without a decrease in their photocatalytic activity, thus showing favorable recycling capability.

## Abstract 2

“Flowering branch”-like PAN-CuS hierarchical heterostructures were *in situ* synthesized through a facile hydrothermal sulfuration growth process on PAN-based fibers prepared by electrospinning.

## Graphical abstract

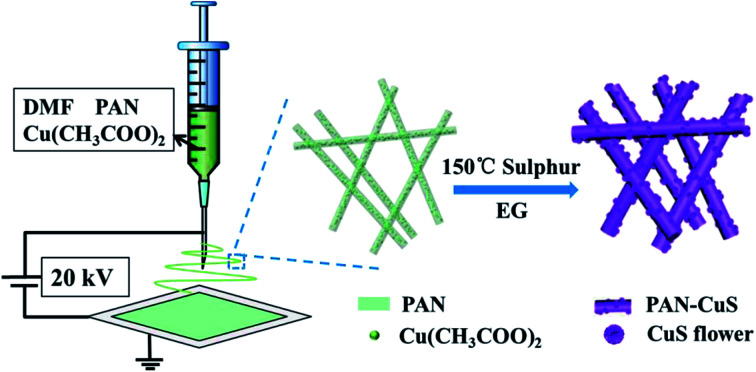

“Green hill and clear water” is not only a “China dream”, but a shared vision of the whole world. As issues related to the environment are drawing increasing attention, many researchers have focused on the degradation of pollutants in industrial wastewater.^1–3^ Dyestuff wastewater accounts for a large proportion of industrial wastewater, which is featured by complicated organic matter composition, high density and toxicity.^4–6^ In recent years, dye degradation technologies have been developed, mainly including adsorption, ozonation, and electrochemical and photochemical degradation.^7–9^ Physical methods cannot resolve the problem extensively and are apt to cause secondary pollution. Biochemical methods have high selectivity, but their technology is complex. Through photocatalytic oxidation, the organic macromolecular pollutants can be oxidized directly or indirectly to non-polluting molecules, such as CO_2_ and H_2_O.^10,11^ This technology also has the advantages of mild operating conditions, significant degrading effects, thorough purification, and a lack of secondary pollution. Photocatalytic oxidation is expected to be one of the most effective treatments. Many previous studies have shown that metal oxide/chalcogenide semiconductors play an important part in the photocatalytic process.^12–16^ However, for now, there are still challenges regarding the large-scale construction of recyclable photocatalytic materials through mild methods.

Metal oxide/chalcogenide semiconductors have the outstanding attributes of unique optical, electronic, magnetic and thermal properties as well as potential applications in energy conversion and catalysis, particularly in the field of photocatalysis.^17–20^ Recently, copper sulfides have attracted a great deal of attention owing to their widespread applications in solar cells, optical filters, photoelectric transformers, superconductors and sensors.^21–23^ Particularly, covellite (CuS) has a strong p-type metallic character with the highest concentration of free carriers among copper sulfide materials.^24^ Many previous researchers have demonstrated that CuS has important applications for photocatalysis. For instance, Saranya *et al.* prepared a CuS catalyst to treat organic pollutants, showing a degradation time of over 60 min.^25^ Thuy *et al.* reported CuS and CuS/ZnS core/shell nanocrystals for photocatalytic degradation of dyes under visible light, and showed that the pollutants almost bleached over a period of 2 h.^26^ Varieties of CuS micro- and nanostructures.^27–35^ have been synthesized using different methods including solid-state synthesis, solvothermal solution based methods, sacrificial templating and chemical vapor deposition techniques.^28,30,36–40,44^ Similar to most reports, CuS has mainly been synthesized in powder form, which is not convenient for recycling. For developing recyclable photocatalytic material, it is important to explore the large-sale preparation methods with low cost. It is known that electrospinning is a powerful method to fabricate one-dimensional functional materials with large specific surface-areas, high porosity, good flexibility and high stability.^41–43^

Herein, we elaborately developed stringed “flowering branch” PAN-CuS with hierarchical architecture combining fibrous PAN skeleton and active CuS material. First, we obtained PAN-Cu^2+^ composite nanofibers from a simple electrospinning method. Then, the PAN-CuS composite nanostructures were prepared through a further *in situ* sulfidizing process. Then, the degradation activity for the dye pollutants and recyclability of the as-synthesized PAN-CuS nanostructures were studied. The superior photocatalytic activity can be attributed to the stable fibrilous structure and abundant active sites of the CuS nanoflowers assembled from nanosheets. Fig. 1 illustrates the synthesis process of the PAN-CuS hierarchical heterostructures.

**Fig. 1:**
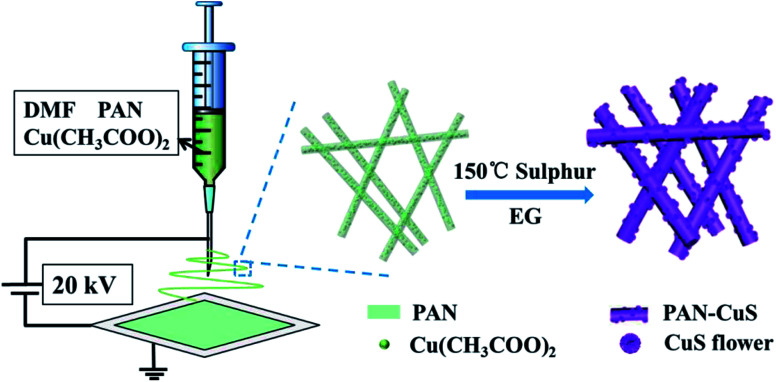
Schematic of the synthesis of PAN-CuS hierarchical heterostructures.

To explore the effects of different amounts of Cu^2+^, 1 mmol, 1.5 mmol, or 2 mmol Cu(CH_3_COO)_2_ was added to PAN/DMF to synthesize PAN-Cu-1, PAN-Cu-2, PAN-Cu-3 nanofilms, respectively. Fig. S1a and b† show the SEM images of the PAN-Cu-2 nanofibers. The nanofiber has a diameter of about 500 nm and length of several tens of micrometers, with a continuous interlacing network. After sulfidation, Cu^2+^ ions move to the surface to produce CuS nanoflowers, and the nanofiber structure is well-maintained to function as the primary structure. As shown in Fig. S1c and d,† the diameter of the CuS nanoflower is about 200 nm, and is assembled from nanosheets, thus possessing abundant active sites. A previous study^44^ reported the formation process as follows:

1 2HOCH_2_CH_2_OH − 2H_2_O → 2CH_3_CHO → 2H_3_CCOCOCH_3_ + 2H

2 S + 2H → H_2_S

3 Cu^2+^ + H_2_S → CuS + 2H^+^.

The reaction temperature was chosen to be 150 °C to exceed the melting point of sulfur (120 °C). First, acetaldehyde can be generated by the dehydration of EG at high temperatures, where acetaldehyde can donate a hydrogen atom and act as a reducing agent, as shown in eqn (1). Then, S^2−^ ions are formed through the reduction of S by H atoms, as shown in eqn (2). When the PAN film came in contact with EG, the Cu^2+^ ions move from the inside to the surface and then form the CuS crystal nucleus. When the ion concentration was moderate, the crystal nucleus grew into nanosheets and further self-assembled into nanoflowers. As shown in Fig. 2a–f, for PAN-CuS-1 and PAN-CuS-2, more Cu^2+^ corresponds to more CuS flowers. However, as Cu^2+^ further increased, the CuS of the PAN-CuS-3 mainly showed the morphology of nanosheets. This phenomenon was proposed to be attributed to the increased CuS nucleation number with high Cu^2+^ content; this will result in a lower crystal growth process, thus limiting the formation of CuS nanoflowers.^45^ In addition, we prepared CuS on pure PAN fibers for comparison. The SEM image is shown in Fig. S2.† Compared with *in situ* synthesis of CuS on PAN-Cu^2+^ fibers, Fig. S2† shows that CuS flowers are very uneven, indicating the advantage of the *in situ* sulfuration method of PAN-Cu^2+^ films.

**Fig. 2:**
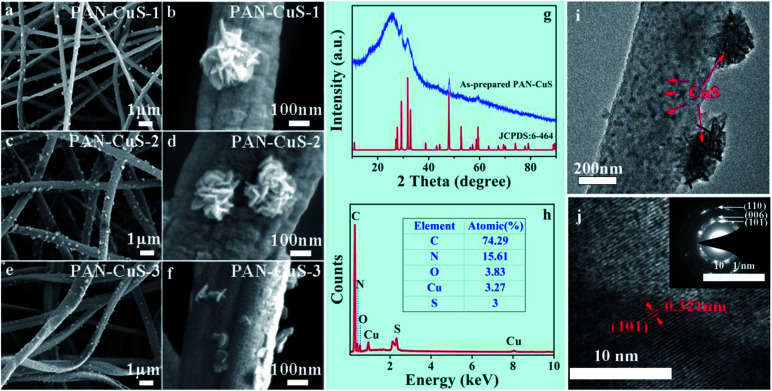
(a–f) SEM images of different PAN-CuS hierarchical heterostructures. (a and b) PAN-CuS-1. (c and d) PAN-CuS-2. (e and f) PAN-CuS-3. (g) XRD diffraction patterns of PAN-CuS-2 hierarchical heterostructures. (h) A typical EDX spectrum of the PAN-CuS-2 hierarchical heterostructures. (i) TEM image of PAN-CuS-2 hierarchical heterostructures; (j) HRTEM image of the heterojunction region and SAED of the nanosheet (inset).

The XRD pattern of PAN-CuS-2 films (Fig. 2g) shows a broad diffraction peak at around 24.9°, which is attributed to the electrospun nanofibers.^46,47^ Other diffraction peaks can be well assigned to CuS (JCPDS no. 6-464). No additional peaks for other phases were observed, indicating the high purity of CuS. The EDX spectrum (Fig. 2h) further confirms the presence of C, O, Cu and S elements in the hierarchical nanostructures. The EDX spectra of PAN-CuS-1, PAN-CuS-3 are also shown in Fig. S3† for understanding the components of these samples. The microstructure was further examined using transmission electron microscopy (TEM). The TEM image in Fig. 2i clearly demonstrates the heterostructure of the PAN-CuS composite. The CuS flower is assembled by nanosheets with an average length of about 50 nm. It is expected that these CuS nanosheets possess relative more active sites. The high magnification TEM image in Fig. 2j shows the lattice pattern of the CuS nanoparticle and reveals a lattice spacing of 0.321 nm, which is in a good agreement with the interplanar distance of (101) of hexagonal CuS. The selected-area electron diffraction pattern (Fig. 2j inset) indicates that the CuS nanoparticles are polycrystalline. As shown in Fig. S4,† FT-IR spectroscopy was performed to identify the components of the nanocomposites. As shown in the FT-IR spectrum, the characteristic peak at 1072.28 cm^−1^ can be attributed to the S=O and C–C stretching vibration, while the vibration peaks at 1359.91 cm^−1^ and 1450.46 cm^−1^ are associated with the CH_3_ and CH_2_ stretching vibrations, respectively. The peak at 1730.24 cm^−1^ can be attributed to the C=O stretching vibration. The peak at 2241.64 cm^−1^ is attributed to the stretching vibration of C≡N. The peaks at 2934.24 cm^−1^ can be attributed to the C–H stretching vibration in CH_3_. Furthermore, the presence of vibrational peaks at 612.71 cm^−1^ indicates the presence of Cu–S stretching modes. The surface chemistry of the as-developed PAN-CuS film was studied by XPS measurement. In Fig. S5a,† the wide-scan XPS spectrum indicated the presence of Cu and S from the CuS nanoparticles as well as C, N, and O from the electrospun PAN nanofibers and other organic solvents. Fig. S5b† represents a high-resolution spectrum of Cu 2p. The peaks for Cu indicated the typical Cu 2p3/2 (932.30 eV) and Cu 2p1/2 (952.80 eV) binding energies. The XPS spectrum of S 2p in Fig. S5c† is characterized by peaks at 162.5 and 163.2 eV. At the same time, the C peak centered at 285 eV could be assigned to the bonds of C–C (284.6 eV), C–O (285.6 eV), and O–C=O (289 eV), demonstrating the presence of the carboxyl carbon.

We explored the catalytic properties of different samples: pure PAN film, PAN-Cu^2+^ film, and PAN-CuS film. All the tests were conducted in the presence of H_2_O_2_ under UV light.

Fig. 3a shows the absorption spectra of aqueous solutions of MB tested at different intervals in the presence of the CuS (PAN-CuS-2) architectures. The intense absorption peak at 664 nm of MB decreased gradually with the prolonged irradiation time, indicating the degradation of MB. The decoloring degree of aqueous MB reached 46.2%, 80.7%, 93.6%, and 97.1% after 15, 30, 45, 50 min, respectively. This good catalytic performance can be attributed to the unique features of the hierarchical nanostructures: fibrous skeleton, transition metal sulfide, abundant active sites, high porosity and strong connection between CuS and electrospun nanofibers. When H_2_O_2_ was added, photoelectrons were consumed, thus preventing photonic electrons from recombining with holes, which improved quantum efficiency. Fig. 3b exhibits the degradation activity for different Cu content. For PAN-CuS-1, the decoloring degree was 37.6%, 77.0%, 92.4%, and 94.6% in 15, 30, 45, 50 min, respectively. For PAN-CuS-3, the decoloring degree was 57.9%, 86.1%, 94.6%, and 96.7% in 15, 30, 45, and 50 min, respectively. As the Cu content increased, the degradation rate increased. To further study the degradation kinetics, the first order rate constants for the degradation of MB (Fig. 3c) were calculated to be 0.07032 min^−1^, 0.08035 min^−1^, and 0.07099 min^−1^. The first order rate constant is described as follows:ln(*C*_0_/*C*_*t*_) = *kt*

**Fig. 3:**
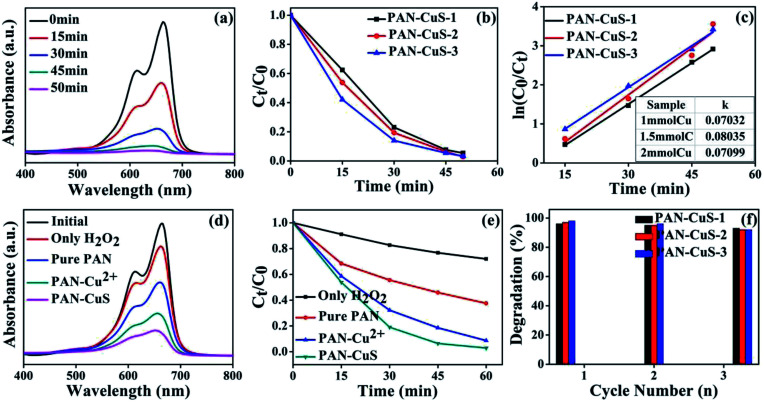
(a) UV-Vis absorption spectra of MB solutions for different durations with PAN-CuS-2. (b) The MB degradation rates for different amounts of Cu^2+^. (c) Kinetic study for the degradation of MB; the inset shows the first order rate constant. (d) UV-Vis absorption spectra of MB degradation after 30 min for different samples: only H_2_O_2_, pure PAN, PAN-Cu^2+^, PAN-CuS. (e) The MB degradation rates of different samples: only H_2_O_2_, pure PAN, PAN-Cu^2+^, PAN-CuS. (f) The % degradation *vs.* cycle number suggesting the stability of PAN-CuS hierarchical structures toward MB degradation. All the measurements were carried out under UV.

where *k* is the apparent rate constant, *C*_0_ is the original concentration of MB, and *C*_*t*_ is the equilibrium concentration of MB at the relative reaction time. The calculated results show that the PAN-CuS-2 film has the highest *k* among the samples. As mentioned above, more CuS nanoflowers lead to better catalytic properties, indicating that CuS indeed plays a decisive role. Fig. 3d systematically displays MB degradation activity after 30 min for different samples. When adding only H_2_O_2_ without PAN-CuS catalyst, the degradation of MB was only 17%, and the pure PAN film also had a mild effect on MB due to its adsorption capacity (the degradation degree was about 45%). However, the pure PAN film corroded after one cycle and could not be reused. For PAN-Cu^2+^ and PAN-CuS, the degradation of MB was 70% and 83%, respectively, which demonstrates the advantage of the *in situ* sulfuration method of PAN-Cu films. Fig. 3e also displays the MB degradation activity by plotting *C*_*t*_/*C*_0_ as a function of time; the samples were the same as those in Fig. 3d. Thus, the good photocatalytic performance of PAN-CuS hierarchical heterostructures is due to the following factors. First, the nanosheet structure of CuS not only allows more surfaces to receive the incident light, but also exhibits more active catalytic sites, which results in a good photocatalytic performance. Second, the as-adopted fabrication route successfully realized close contact between the CuS nanosheets and the PAN fibers in the heterostructures. Such close contact is more effective for suppression of electron–hole recombination. Third, this PAN-CuS film can be easily recycled.

Recycling as well as maintaining high photocatalytic activity is a critical issue for the long-term use of catalysts in practical applications. Consequently, two factors need to be considered.^46^ (1) The ability of the catalyst to maintain its high activity over time is critical. It is known that the photocorrosion or photodissolution of photocatalysts may occur on the surface during the photocatalytic reaction. To test the stability of MB photodegradation on PAN-CuS films, we reused the catalyst three times. As shown in Fig. 3f, each experiment was performed under identical conditions and after three cycles, the photocatalytic activity of the PAN-CuS hierarchical heterostructures remained 94%. (2) The ease with which the catalyst can be separated from solution must also be considered. In this study, the samples are nanofibrous and the films can be directly removed from the solution. Fig. S6† shows the SEM images of the samples after three cycles.

For studying the universality of the as-prepared PAN-CuS photocatalytic material, PAN-CuS-2 was also used to degrade the Rhodamine B (RhB) dye. The degradation result is exhibited in Fig. 4 (left). The decoloring degree was 53.7%, 86.1%, 93.9%, and 98.4% after 10, 20, 30, and 40 min, respectively, indicating excellent degradation activity. On the basis of the above results, the dye degradation mechanism^48^ can be ascribed to the formation of hydroxide radicals in the presence of CuS and H_2_O_2_. The hydroxyl radicals, in turn, react with and degrade organic substrates such as RhB. Fig. 4 (right) shows the schematic of RhB degradation in the presence of H_2_O_2_ under UV light.
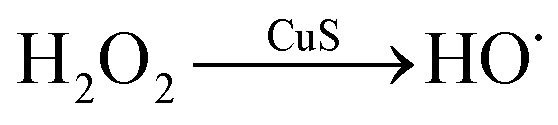
RH + HO˙ → R˙ + H_2_O (RH organic substrates such as RhB)

**Fig. 4:**
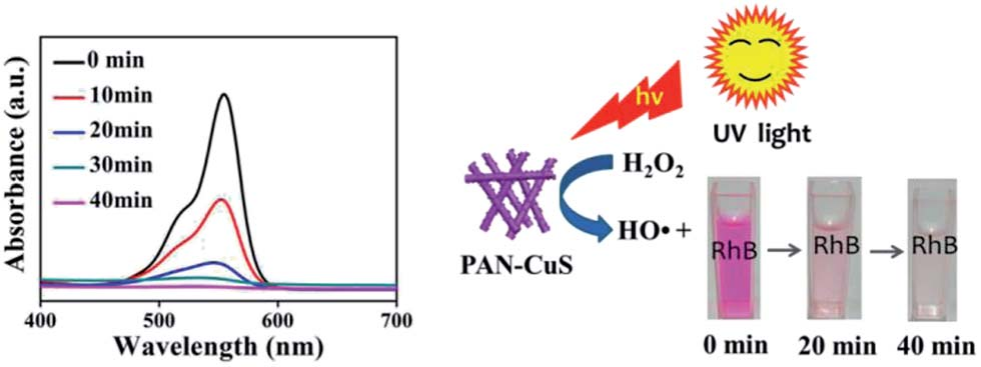
(Left) UV-Vis absorption spectra of RhB solutions at different durations with PAN-CuS-2, (right) schematic of RhB degradation as a function of time after adding 1 mL of H_2_O_2_ with PAN-CuS hierarchical heterostructures under UV.

## Conclusions

In summary, stringed “flowering branch” PAN-CuS hierarchical heterostructures were successfully fabricated through *in situ* sulfidation on electrospun fibers. This unique structure can provide abundant active sites, controllable morphology, and excellent charge separation, thus exhibiting enhanced photocatalytic activity in the decomposition of MB and RhB under UV light irradiation. Furthermore, these PAN-CuS hierarchical heterostructures with good flexibility can be easily recycled without a decrease in the photocatalytic activity. The results show that PAN-CuS-2 film has the strongest degradation effect, highlighting the role of CuS nanoflower assembly of nanosheets. It is expected that the development of such nanofibers by electrospinning with hierarchical heterostructures represents a very simple and cost-effective approach for the degradation of dye and will greatly promote their practical application to eliminate organic pollutants from wastewater.

## Conflicts of interest

There are no conflicts to declare.

## Supplementary Material

RA-008-C8RA08293F-s001
