# Supplementary material for: In situ sulfuration synthesis of flexible PAN-CuS “flowering branch” heterostructures as recyclable catalysts for dye degradation
Source: RSC Adv. 2018 Dec 5;8(71):40589–94. doi: 10.1039/c8ra08293f (PMC9091420; doi:10.1039/c8ra08293f)
Supplement: RA-008-C8RA08293F-s001 [file RA-008-C8RA08293F-s001.pdf]

Electronic Supplementary Material (ESI) for RSC Advances.
This journal is © The Royal Society of Chemistry 2018

Supporting Information

In Situ Sulfuration Synthesis of Flexible PAN-CuS "Flowering Branch" Heterostructures as Recyclable Catalyst for Dye Degradation

Yin Lu, [a] Yanjie Wang, [a] Shizhong Cui, [a] Weihua Chen, [*b] and Liwei Mi [*a]

[a]*Center for Advanced Materials Research, Zhongyuan University of Technology, Zhengzhou, 450007, P.R. China. E-mail address: mlwzzu@163.com.*

[b]*College of Chemistry and Molecular Engineering, Zhengzhou University, Zhengzhou, 450001, P. R. China. E-mail address:chenweih@zzu.edu.cn.*

Experimental section

1. Chemicals.

Polyacrylonitrile (PAN, Mw = 150 000 g mol^{-1}), cupric acetate anhydrous($Cu(CH_3COO)_2$), N,N-dimethylformamide (DMF), ethylene glycol (EG), sulfur powder (S), hydrogen peroxide (H_2O_2, 30%). All chemicals were of analytic grade and used without further purification.

2. Preparation of the PAN-Cu nanofibers

PAN-Cu precursor nanofibers were prepared by a common electrospinning method. In a typical process, 0.10 g of PAN was dissolved in 10ml DMF at 70 °C with oil bath for hours under magnetic stirring. Then $Cu(CH_3COO)_2$ was added into the intermixture, still with magnetic stirring. Next, the mixture was transferred to a plastic needle at a constant flow rate of 1ml h^{-1} and electrospun at 20 kV. The collector-to-needle distance was maintained at 16 cm to collect the nanofiber film on an aluminum foil. In purpose of comparison, the pure PAN fabric was also prepared under the same conditions by using blank PAN/DMF solution.

3. Fabrication of PAN-CuS nanofiber hierarchical heterostructures

PAN-CuS composites were prepared by a solvothermal process. In a typical procedure, 1.5 mmol S was added to 10ml EG, after stirring for hours, the as prepared electrospun carbon nanofibers PAN-Cu-1, PAN-Cu-2, PAN-Cu-3 were dispersed in the mixed solution, respectively, then the mixture was transferred into a Teflon-lined stainless steel autoclave with a capacity of 30 mL. Subsequently, the autoclave was sealed and maintained at 150 °C for 10 h. After solvothermal reaction, the autoclave was air-cooled to room temperature. The films were collected, washed with ethanol and deionized water several times, and finally dried at 80 °C for 4 h. PAN-CuS-1, PAN-CuS-2, PAN-CuS-3 were obtained. For comparison, the pure PAN film was put in a mixed solution, which contains 2mmol $Cu(CH_3COO)_2$, 1.5mmol S and 10ml GE. The reaction condition was the same with PAN-CuS(150 °C for 10 h).

4. Characterization.

X-ray diffraction (XRD) patterns were obtained with a Bruker D8 Advance X-ray powder diffractometer using Cu-Ka irradiation at a scan rate of 0.1^{o} s^{-1}. All XRD measurements were performed within $10^{o} \leq 2\theta \leq 90^{o}$ at room temperature. The surface morphology of as-prepared products was examined with a field-emission scanning electron microscope (FESEM, Merlin Compact, ZEISS) equipped with an energy dispersive X-ray spectroscopy (EDX) system. Transmission electron microscopy (TEM) and high-resolution TEM (HRTEM) observations were recorded by a Tecnai G2 F20 S-TWIN (operated at 200 kV). The functional groups were studied by FT-IR spectroscopy (Nicolet iS50). X-Ray photoelectron spectroscopy (XPS) spectra was recorded on a Thermo ESCALAB

250Xi. The X-ray photoelectron spectrometer using non-monochromatized Al-Ka X-rays as the excitation source. The optical property of CuS samples was measured with a Perkin-Elmer Lambda 950 UV/VIS/NIR spectrophotometer.

5. Catalytic Degradation of MB using PAN-CuS hierarchical heterostructures.

The catalytic activity of different as-synthesized self-assembled CuS films were studied by MB degradation. 1 mL of H_2O_2 and catalyst film(2cm×2cm) were dispersed in 20 mL 20mg L^{-1} MB solution, and the solution was stirred in the UV-light for different durations. The MB solution was collected after selected durations, and the MB concentration was measured with a PerkinElmer Lambda 950 UV/VIS/NIR spectrophotometer.

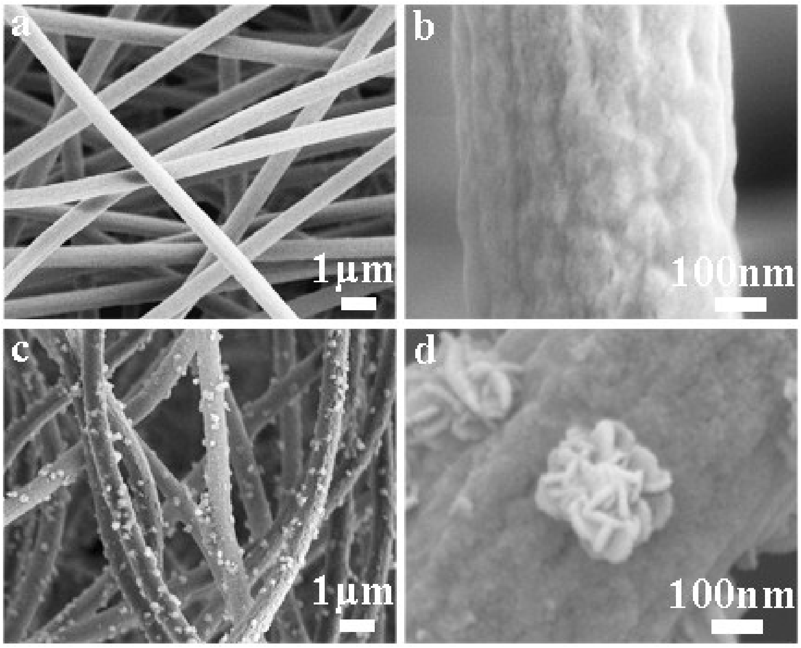

Fig.S1 SEM images of as-prepared samples. (a,b) PAN-Cu^{2+}. (c,d) PAN-CuS

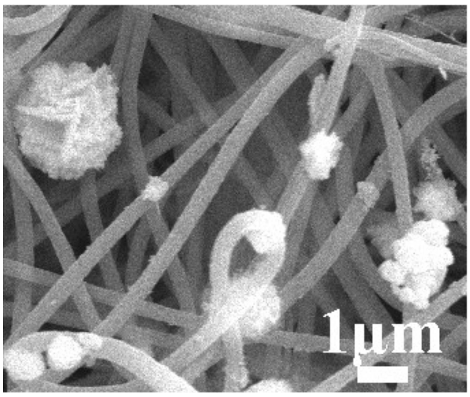

Fig. S2. SEM images of comparative sample of CuS on pure PAN fibers.

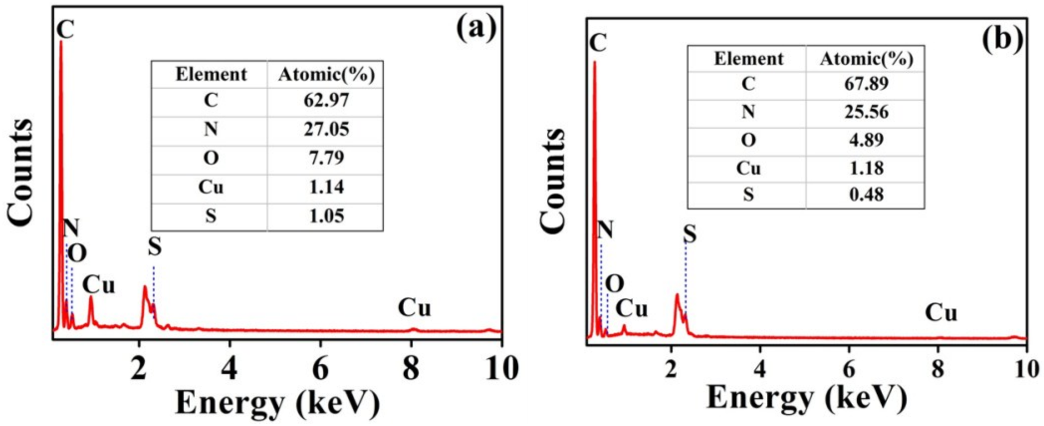

Fig.S3. EDX spectrum of PAN-CuS-1, PAN-CuS-3

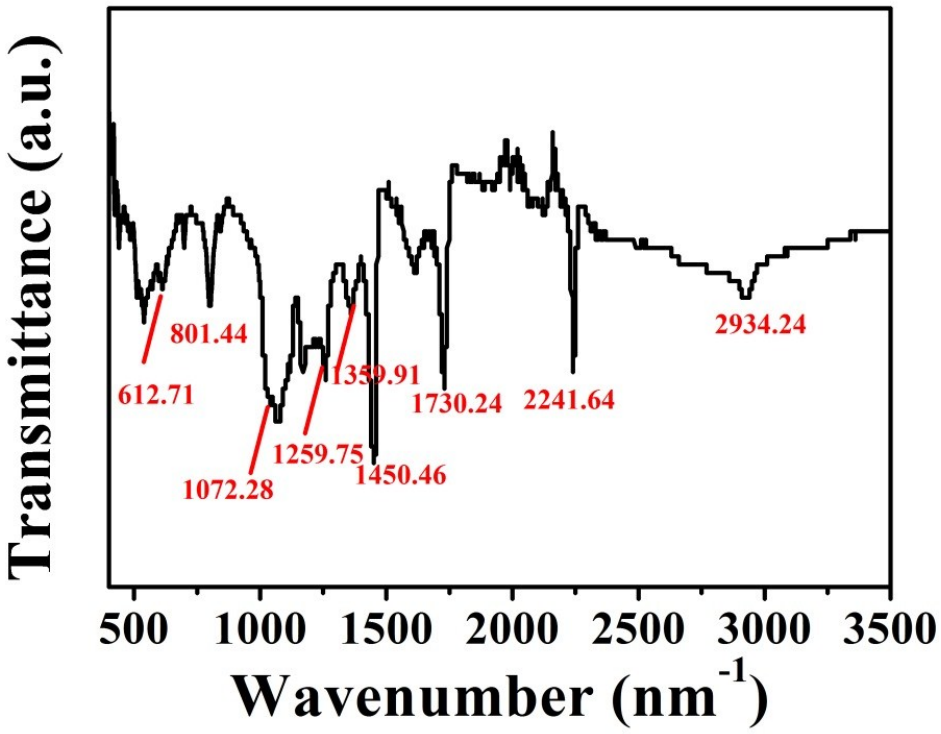

Fig.S4. FT-IR spectra of PAN-CuS-2

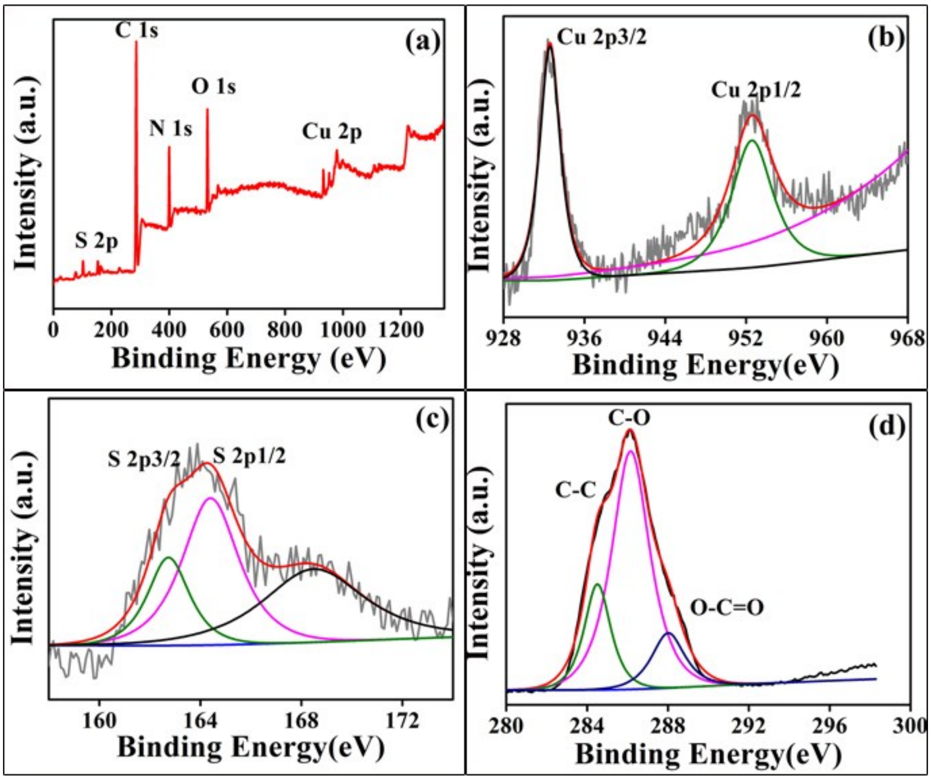

Fig.S5 (a) XPS fully scanned spectrum of the PAN-CuS-2 hierarchical heterostructures. (b) Cu 2p peak. (c) S 2p peak. (d) C 1s peak in the composite

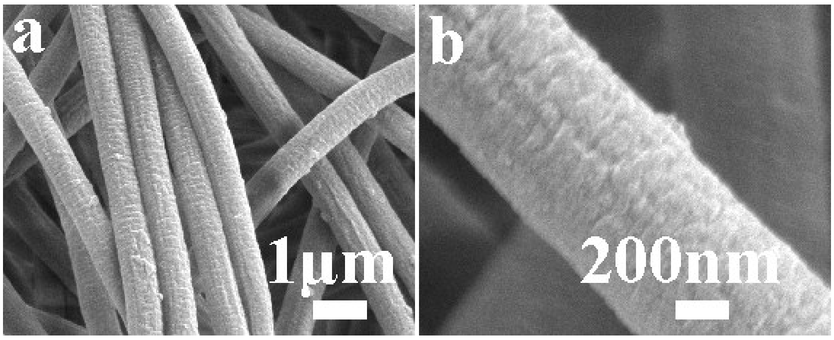

Fig.S6. SEM images of PAN-CuS after three times recycle.
